# Community engagement to improve access to healthcare: a comparative case study to advance implementation science for transgender health equity

**DOI:** 10.1186/s12939-022-01702-8

**Published:** 2022-07-31

**Authors:** Hale M. Thompson, Allison M. Clement, Reyna Ortiz, Toni Marie Preston, Ava L. Wells Quantrell, Michelle Enfield, A. J. King, Lee Klosinski, Cathy J. Reback, Alison Hamilton, Norweeta Milburn

**Affiliations:** 1 Rush University Medical Center, 1645 W. Jackson Blvd., Suite 302, Chicago, IL 60612 USA; 2 University of California-Los Angeles, 760 Westwood Plaza A8-159A, Los Angeles, CA 90095 USA; 3 TaskForce Prevention & Community Services, 9 N. Cicero, Chicago, IL 60644 USA; 4 Howard Brown Health Center, 4025 N. Sheridan Road, Chicago, IL 60613 USA; 5 AIDS Project Los Angeles, 5901 W. Olympic Blvd., Suite 310, Los Angeles, CA 90036 USA; 6 Center for AIDS Research, University of California-Los Angeles, 11075 Santa Monica Blvd, Suite 100, Los Angeles, CA 90025 USA; 7 University of California-Los Angeles, 760 Westwood PlazaA8-159A, Los Angeles, CA 90095 USA; 8 Friends Research Institute, 6910 Santa Monica Boulevard, Los Angeles, CA 90038 USA; 9 University of California-Los Angeles, 760 Westwood Plaza, Los Angeles, CA 90095 USA

**Keywords:** Transgender, Community engagement, Health equity, EPIS framework, Comparative case study, World Café model, Human-centered design

## Abstract

**Background:**

Recent calls to action have been made for Implementation Science to attend to health inequities at the intersections of race, gender, and social injustice in the United States. Transgender people, particularly Black and Latina transgender women, experience a range of health inequities and social injustices. In this study, we compared two processes of transgender community engagement in Los Angeles and in Chicago as an implementation strategy to address inequitable access to care; we adapted and extended the Exploration Planning Implementation and Sustainment (EPIS) framework for transgender health equity.

**Methods:**

A comparative case method and the EPIS framework were used to examine parallel implementation strategies of transgender community engagement to expand access to care. To foster conceptual development and adaptation of EPIS for trans health equity, the comparative case method required detailed description, exploration, and analyses of the community-engagement processes that led to different interventions to expand access. In both cities, the unit of analysis was a steering committee made up of local transgender and cisgender stakeholders.

**Results:**

Both steering committees initiated their exploration processes with World Café-style, transgender community-engaged events in order to assess community needs and structural barriers to healthcare. The steering committees curated activities that amplified the voices of transgender community members among stakeholders, encouraging more effective and collaborative ways to advance transgender health equity. Based on analysis and findings from the Los Angeles town hall, the steering committee worked with a local medical school, extending the transgender medicine curriculum, and incorporating elements of transgender community-engagement. The Chicago steering committee determined from their findings that the most impactful intervention on structural racism and barriers to healthcare access would be to design and pilot an employment program for Black and Latina transgender women.

**Conclusion:**

In Los Angeles and Chicago, transgender community engagement guided implementation processes and led to critical insights regarding specific, local barriers to healthcare. The steering committee itself represented an important vehicle for individual-, organizational-, and community-level relationship and capacity building. This comparative case study highlights key adaptations of EPIS toward the formation of an implementation science framework for transgender health equity.

## Background & Objective

Implementation science (IS) has grown from a nascent field in the 1990s to a critical twenty-first century discipline that interrogates pathways from efficacious research to the deployment of evidence-based interventions [1]. IS systematically analyzes layers of contexts and a range of stakeholder experiences to determine intervention acceptability, feasibility, and impact in real-world settings. Essentially, IS serves to close the gap between research and practice [2]. In the context of dual pandemics, the COVID-19 pandemic and the pandemic of structural racism and anti-Black violence, a new call to action has been made for IS to focus on health inequities at the intersections of race, gender, and social injustice [3–6]. Building upon this call, we draw specific attention to transgender^1^ health inequity at the intersections of race, ethnicity, and immigration status and the critical importance of community engagement for implementation science.

## Trans health inequity

Transgender communities face enormous social and health disparities in the United States. Transgender women, in particular, experience high levels of poverty, housing instability, food insecurity alongside limited access to employment and healthcare; associated with these social determinants are poor health outcomes related to trauma exposure, substance misuse, depression and anxiety, and HIV compared to other adult populations [7–12]. At the intersections of race, citizenship, and gender, the disparities widen for Black, Latina/x^1^, Native American, and immigrant transfeminine populations, and these intersectional categories may compound barriers to care [7, 12–14]. In the domain of HIV, 19% of Black transgender women reported living with HIV, compared to 1.4% of all respondents in the 2015 US Transgender Survey [7]. In a 2019 – 2020 National Health Behavior Survey of 1,608 transgender women, 42% had a valid positive test for HIV, with the highest prevalence among Native American (65%), Black (62%), and Latina/x (35%) [14].

The Los Angeles Department of Public Health estimated that there were over 14,000 transgender individuals living in Los Angeles County; they also estimated that over 15% of transgender women were living with HIV (Los Angeles Department of Public Health, 2012). The UCLA Williams Institute surveyed nearly 400 transgender women living with HIV in Los Angeles County and found that 44% experienced challenges accessing health care in the previous year. More specifically, 67% could not get medication, and 47% could not get medical care when they needed it [15].

The Chicago Department of Public Health estimated that there were 10,500 transgender adult residents in the city, or 0.05% of the adult population [16], and yet transgender women make up 2% to 2.6% of the newly diagnosed HIV cases in 2017 and 2019, respectively [17]. A 2016 HIV-positive cohort of transgender women in Chicago – 94% Black, 5% Latina/x, with a mean age of 30 years—indicated the following baseline characteristics: 94% were currently unemployed, 84% had an annual income < $6,000, and 77% had experienced homelessness as an adult [18]. To address ongoing systemic marginalization and harm, the research team concluded that design and implementation of effective structural health interventions require enhanced efforts and strategies for transgender community engagement [18].

To our knowledge, very few transgender health or HIV prevention intervention studies have used community engagement and IS frameworks to identify elements key to transgender health equity. City-, state-, and national-level transgender health and HIV needs assessments have engaged transgender community members for study design, recruitment, and data collection [7, 19–24]. Drawing on health services research, Wolfe and colleagues recognized the critical role transgender veterans played in their research and recommended continued community engagement to inform the health services research agenda regarding gender-affirming care in the Veterans Health Administration network [25]. Similarly, Wesp and colleagues built on the works of Kimberle Crenshaw, Dean Spade, and Eric Stanley to propose a conceptual framework for structural analysis of trans health inequities; their applications of the framework focused on research itself rather than intervention implementation and impact [26].

## Comparative case study to advance a transgender health equity IS framework

The case study method serves many key purposes in qualitative research. Through descriptive and interpretive recontextualization [27], the case study registers the importance of the case and its contextual conditions for impact on health outcomes [28]. More broadly, the case may serve as both a benchmark and horizon for what is possible for other cases [27]. The case study method enables exploration of concepts that cannot easily be quantified, measured, or validated, and, historically, has contributed to understanding urban life and the integration of the social ecological resources, rejecting empirical assumptions and refusing to accept what is visible as social reality [29]. This critical approach to community health practice and research uses conceptual and theoretical interventions to extend more traditional health research methods [30], fostering theory development, in this case a trans health equity IS framework, through detailed description, exploration, and analyses of a social phenomenon like community engagement [31].

Unlike with grounded theory methods, experiences articulated by transgender community members are not the point of departure for theory. Theoretical frameworks—explored via case study—aim to identify the logics that undergird the social realities that participants may express [29]. Cases operate in specific, local contexts and provide a window onto broader social phenomena. At case-specific intersections of structural racism, sexism, nationalism, economic marginalization, and transphobia, we have adapted an IS framework to identify unique barriers and facilitators to trans health intervention implementation [3, 4, 32–35].

### Objective

In this study, our objective was to highlight community engagement as an exemplary strategy to advance IS for transgender health equity. We use a comparative case study method and the Exploration, Preparation, Implementation and Sustainment (EPIS) framework to explore two recent examples, one in Los Angeles and one in Chicago, of transgender community-engaged implementation practice for expanding access to quality care and improving health equity [29, 33]. Our comparative case study enabled us to identify contextual factors and processes of community engagement that have facilitated multilevel interventions to address transgender health equity. Based on our findings, we extended and adapted the EPIS framework into an intersectional one—a trans health equity IS framework—that accounts for and addresses transgender community engagement at the core of health equity.

## Methods

### Settings and case definitions

Following Baxter and Jack, our cases are defined by the following parameters [28]. First, these cases originated in 2016 and 2019 in urban U.S. settings – Los Angeles and Chicago, specifically – and are ongoing. Second, they both began as community-engagement projects with the goal of creating and implementing an intervention that would expand access to care for transgender people and, in Chicago, for Black and Latina transgender women, specifically. For both cases, the community engagement process was planned, driven, and analyzed by a steering committee consisting of a cross-section of stakeholders and community members spanning a range of ages, genders, races, ethnicities, organizations, and professions. In 2022, the Trans Accountability Project (TAP) steering committee began its fourth year, and the LA steering committee has not convened since the pandemic began in 2020. As members of these steering committees, the author team acknowledges unequal power dynamics within each steering committee despite attempts to level them through these collaborations; these dynamics are emblematic of both the inequitable distribution of access to resources that transgender communities face as well as the need for trans health equity implementation frameworks.

In Los Angeles, the steering committee consisted of a combination of cisgender and transgender stakeholders who were primarily employed as faculty and health researchers, program staff, trainers, and/or advocates at UCLA, UCLA Health, and local community-based organizations that provide healthcare, services, and conduct health research with transgender individuals. There were approximately ten members at any given time while the project was ongoing, with representation from Latinx, Black, Native American, and White communities. The Los Angeles steering committee met on several occasions leading up to the main community event (referred to as the “Town Hall Meeting”) in order to determine the location, the format, and logistics of the event, brainstorm discussion questions, and assign facilitator and notetaker roles.

The steering committee was also critical to the next steps of the project. Immediately following the Town Hall Meeting, it was responsible for compiling and analyzing the notes from the discussions and authoring the recommendations based on the analysis. Meetings were then convened on a quarterly or biannual basis to enable the steering committee to provide ongoing guidance and feedback on next steps throughout the intervention development and implementation process. Ongoing support for the steering committee and its activities was provided by the California HIV/AIDS Research Program through the Center for AIDS Research Health Disparities Core, with additional supplementary funding from the UCLA AIDS Institute.

In Chicago, except for one queer program evaluation expert during year one, the Trans Accountability Project (TAP) steering committee consisted of approximately ten multi-generational and explicitly transgender and nonbinary persons who are public health, housing, and social service providers, advocates, and one faculty researcher. TAP consists of Latinx, Indigenous, Black, White, and multiracial members. Organizationally, TAP is led by the Midwest’s largest FQHC serving LGBTQ + persons and has partnered with three community-based organizations that serve transgender women, particularly in areas with high HIV incidence or prevalence in Chicago. All three partner organizations are small, grassroots, and have significant Trans, Black and Latina leadership.

In addition to its core value of accountability, the TAP steering committee’s overall objective, outlined by the grantor’s funding stream, differed slightly from the Los Angeles objective. TAP’s goal was to mobilize Chicago’s communities of Black and Latina trans women toward the creation of an intervention to address structural racism and expand access to HIV prevention and care. In year one, the objective was to design and conduct a community needs assessment during two mobilization events where community members and allied stakeholders could convene and collectively assess community needs. Based on the findings from the assessment [36], TAP would develop an intervention to address unique, intersectional forms of structural racism and marginalization that hinder access to care for Black and Latina transgender women [35, 37].

### The EPIS framework for trans health equity

Originally developed in a publicly funded, community setting, EPIS offers both flexibility and complexity for evaluating transgender community engagement as an implementation strategy [33, 34, 38]. The EPIS framework specifies four key phases and three key constructs to guide the implementation process. To describe the results of both cases, we use the key constructs, primarily focusing on Bridging and Innovation, plus the Outer Context and Inner Context, to organize and describe the phases of exploration, planning, and implementation. The sustainment phase is largely addressed for future consideration in the discussion as neither project had moved beyond implementation at the time of this analysis. Woodward and colleagues recently advanced work on incorporating health equity domains and an “equity lens” into existing IS frameworks, including EPIS. They specifically recommend integrating culturally relevant factors of recipients, clinical encounter or patient-provider interaction, and societal context. They note that some recent work is focused more on equity in relation to implementation strategies but point out that there is “considerably more work to be done on this…” [39]. We saw this application of an equity lens to be an important opportunity for advancing transgender health equity using community engagement strategies. Our results are organized in terms of the EPIS constructs: Bridging & Innovation followed by the Outer and Inner Contexts.

### Author team, reflexivity, and ethical issues

The author team consists of members of both steering committees. Though the majority of the author team is transgender and/or a person of color, those of us employed directly by academic institutions are primarily cisgender and/or white. Addressing this kind of unequal distribution of power is a focus of both this manuscript and each of the steering committees since power dynamics relate to structural sources of health inequities and health injustice. Though our findings are not generalizable, the author team used the Standards in Reporting Qualitative Research to improve the transparency of our processes [40]. The institutional review boards for each steering committee waived the respective intervention planning, development, and implementation as human subjects research.

## Results

### Bridging & innovation: transgender community engagement to explore, plan, and implement

#### Adapting the World Café model during exploration phases

Developed by Junita Brown and David Isaacs in the 1990s, the World Café model (WCM) provides a platform to bring marginalized voices to discussions around particular civic issues, such as expanding access to care, and facilitates community-building and engagement [41, 42]. Typically, world café events are structured around numerous small-group discussions on curated topics and questions, relevant to specific goals. WCM offers a more horizontal approach to consider health inequities compared to individually focused and extractive methods such as surveys or even focus groups. Put another way, the approach provides a platform for direct communication, relationship building, and multi-directional knowledge sharing among community members and stakeholders whose power may vary with respect to the issue of interest. This approach was selected to generate dialogue around key topics that centered trans voices. Held in safe, accessible spaces reserved specifically for these events, use of WCM promoted cooperative, practical ways of knowing, and connectedness among communities and stakeholders [43].

In Los Angeles and Chicago, both steering committees curated activities that would amplify the voices of transgender community members among stakeholders, encouraging more effective and collaborative ways to advance transgender health equity. Both steering committees used WCM as a method for organizing and developing a foundation upon which to build both relationships and interventions within and across HIV prevention, healthcare, and social service stakeholders and transgender communities.

### Los Angeles: community engagement to inform intervention development

#### The Los Angeles Transgender Town Hall

The Town Hall was hosted at a central Los Angeles location on a weekday evening. Complimentary dinner was served, and gift cards were provided to all Town Hall participants. Over 40 transgender and gender non-conforming participants attended and contributed to the Town Hall event. Using the WCM format, discussions were structured around three areas of concern: (1) primary health care, (2) mental health care, and (3) HIV prevention and treatment. Participants rotated to three different tables for 25 minutes per topic. This rotation afforded each participant the opportunity to provide input across all three domains with different facilitators and peers in each rotation. For each topic, a facilitator used a set of structured probes to prompt conversation in the given subject area, and an observing note-taker recorded participants’ responses and ideas. Groups were available for both English- and Spanish-speaking participants. At the end of the Town Hall, all participants reconvened as a larger group and were able to share their feedback about the event and suggestions for next steps.

#### Planning phase: The Transgender Town Hall findings

After the Town Hall, the steering committee compiled the discussion notes, identified themes, and then developed them into a set of recommendations. Recommendations were organized based on available resources and capacity of the steering committee. For example, regular statewide competency trainings on transgender health and gender-affirming care for organizations funded by the California Department of Public Health, while an appropriate response to the Town Hall findings, would require immense financial and labor resources and thus were deemed less feasible. The recommendations were distributed to the Town Hall participants and shared with the California HIV/AIDS Research Program, the Center for AIDS Research Health Disparities Core’s funder, with the understanding that the document would not be made public or further shared until an implementation and dissemination plan was developed.

#### Implementation phase: transgender health and gender-affirming care curriculum for UCLA Health and the UCLA David Geffen School of Medicine

One of the most prominent findings from the Town Hall was how challenging it was for transgender and gender diverse community members to find sensitive, knowledgeable, and capable health providers. This theme came up across all discussion topics for both English- and Spanish-speaking participants. The lack of competent providers had a direct impact on patient health outcomes, often causing community members to avoid seeking any healthcare at all.

**Fig. 1:**
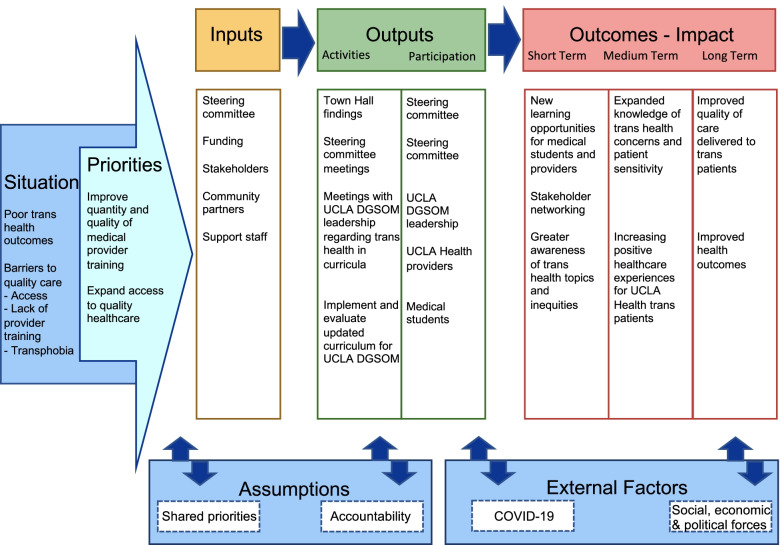
The UCLA CFAR steering committee logic model to develop a trans-affirming medical curriculum

The steering committee determined that based on the recommendations, UCLA was in a unique position to implement change within its health system and school of medicine. They determined that an upstream approach that aimed to promote and enhance the quantity and quality of transgender health education for medical professionals and related issues would be the most effective way to ultimately affect change given limited staffing and funding, with the goal of beginning this process at UCLA and eventually guiding other universities and health systems to incorporate similar approaches. This approach is outlined below and is also reflected in the logic model (see Fig. 1). This plan of action was signed by the steering committee members and was then shared with all of the Town Hall attendees by email, in order to keep participants informed about how their valuable feedback would be used, and as an opportunity to provide comments if so desired. No participant comments were received.

Two UCLA staffers (co-authors AC and LK) who participated on the steering committee and referred to herein as the “project team” met with a range of faculty and administrators to build a collaboration with the UCLA David Geffen School of Medicine (DGSOM) and its leadership. These conversations helped contextualize the curricula that were already being offered related to transgender health and wellness. The project team also became a regular participant in the Clinical Training and Education Workgroup that is part of the UCLA Health System’s LGBTQ Equitable Care Committee. This workgroup is dedicated to the training of UCLA Health staff, residents, fellows, and medical students, and has been an important partnership in advancing the long-term goals of the project.

In 2017 and 2018, as the team ramped up to prepare for the broader DGSOM curriculum intervention, the project team helped to coordinate several brief trainings at UCLA Health to provide expertise on special topics on transgender health (e.g., transgender patient records in electronic health record systems, mental health disparities). During the 2018—2019 academic year, the project team collaborated with UCLA DGSOM to launch an enhanced transgender health session for second-year medical students, building upon existing content of a doctoring class. This session featured a didactic lecture by a subject matter expert, a diverse transgender and non-binary patient panel, and small group discussions between students, faculty, and patient panel members. Evaluation of this updated curriculum demonstrated that it was well-received by students and led to significant changes in knowledge, attitudes, and perceived readiness to work with transgender patients. This session was delivered again to the next two second-year student cohorts during the following two academic years (2019–2020 and 2020–2021). Due to COVID-19, the 2020–2021 session and all of its components were conducted with students virtually via Zoom. Over the course of these three academic years, over 500 medical students participated in the enhanced transgender health curriculum.

Currently, the project team continues to work closely with UCLA DGSOM and its faculty as it rolls out a more thorough and school-wide curriculum redesign. The new curriculum is being rolled out in phases and includes additional LGBTQ health content, including over ten hours of class time dedicated to these topics during the first year of medical school alone. The project team has also been critical in the development, implementation, and evaluation of a pilot elective option for students that focuses exclusively on LGBTQ health and direct experience with LGBTQ patients.

### The Trans Accountability Project in Chicago

#### Exploration: two community conversations & listening sessions

In the first year, the TAP steering committee hosted two community conversations, also known as listening sessions for the stakeholders who attended. Sixty-three community members and ten stakeholders attended the events while nine facilitators from the community and nine notetakers from stakeholder organizations assisted TAP in hosting the events. TAP integrated human-centered design principles into WCM as a framework to curate four different small-group activities, across sixteen groups, and two collective “report-back” discussions that followed the activities. All materials were translated into Spanish, and one table at the first event was conducted in Spanish with two community members.

#### Planning based on findings

Based on TAP’s analyses of the data, five key insights were generated [36]. After much discussion, the steering committee determined that employment would be the domain through which to focus intervention development for years 2 and 3. One element of the intervention would consist of a more downstream approach to improve and expand job-seeking and retention skills of Black and Latina transgender women. The other element would target stakeholders and a more upstream approach to expand employment opportunities and working environments for Black and Latina transgender women. At the same time each partner organization would expand its own particular employment services that were enhanced by TAP funding.

#### The COVID-19 pivot

**Fig. 2:**
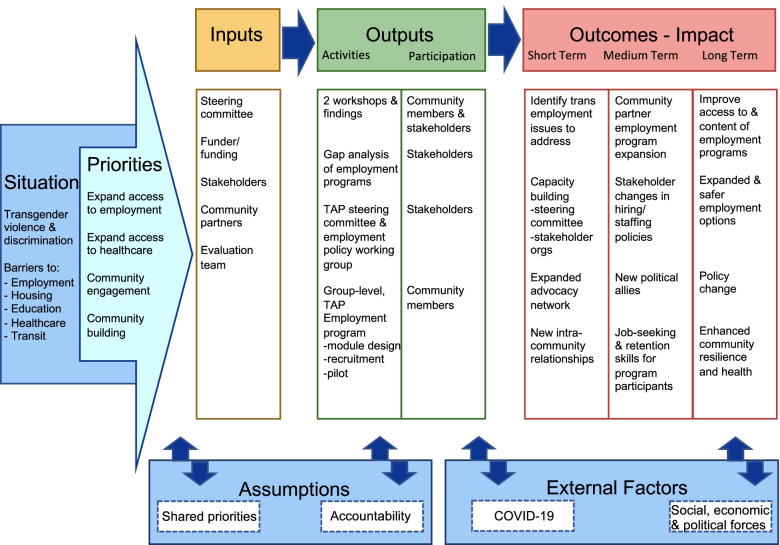
Trans Accountability Project (TAP) logic model as a multi-level employment intervention, including the group-level TAP Employment Program

Year two of TAP consisted of setbacks in the development of the intervention due to onset of the global COVID-19 pandemic. The steering committee began year two with the development of an employment intervention logic model (see Fig. 2) and a scan of local employment interventions accessible to Black and Latina transgender women. With the pandemic, however, partner organizations shifted the majority of programming from in-person to virtual formats in order to minimize staff and client exposure, expand access to COVID-19 testing, and also to ensure services continued to reach community members during mandated closures of community spaces.

#### Implementation: the TAP Employment Program and stakeholder engagement

In year three, the TAP Steering Committee completed the design of the employment intervention and launched the pilot. The pilot consisted of eight, two-hour monthly modules with a corresponding monthly speaker series; in total, there were 16 Zoom sessions on eight employment-focused topics. In the first month’s module, a visioning activity was conducted with participants to discuss their dream jobs. The first speaker was an elder and leader in Chicago trans communities who had been conducting HIV prevention for over 30 years. She also talked about what her work had been like during COVID-19 and how she maintained accessible HIV testing. In month seven, participants focused on job applications and engaged in mock interviews.

With limited recruitment efforts, due in part to COVID-19, five participants ranging in age, race and ethnicity, transition status (i.e., various combinations of legal, social, and medical transition) as well as geographic locations, enrolled and attended the pilot virtually. The TAP steering committee members typically attended each session, contributing to the activities and discussions, as well as the speaker series. These sustained group meetings between participants and steering committee members, plus speakers from the community, helped create a supportive network for the participants in terms of finding and retaining employment.

Partner organizations represented by steering committee members also used TAP funding to hire additional staff and provide employment services to community members not enrolled in the program. One organization, for example, has an extensive clothing closet and cosmetic supplies. Another partner organization conducts outreach with Chicago-area employers to assess and identify ways to improve their capacity to provide safe and affirming workplaces for Black and Latina transgender women.

On the advocacy front, stakeholders are periodically engaged to prioritize collectively any organizational, local, and state policies that may help transform employment or healthcare barriers into facilitators. TAP steering committee members and stakeholders participate in ongoing workgroups to change policies and advance state legislation around background checks, name changes, as well as the decriminalization of sex work. A current legislative priority, for example, is Illinois House Bill 2542, also known as the name change modernization bill. With passage, this law would simplify the process for legal name changes in general, waive the ten-year waiting period required of persons with felony convictions, and repeal the ban for persons convicted of identity theft. The existing law has prevented thousands of trans persons in Illinois from legal name change which creates additional employment barriers [44]. In 2021, the Illinois House passed the bill but did not pass in Senate Committee. In 2022, despite growing support among Senators, the bill has stalled.

#### Outer Context: federal policy, funding, and a global pandemic

Broadly, the outer contexts for the two cases are similar; only the funding sources and scales differed. Notably, the social and political contexts from which funding is derived shape the processes of exploration, implementation, and sustainment of innovative interventions [34]. Considering U.S. federal healthcare policy as a shared outer context of both cases, the Affordable Care Act (ACA), Sect. 1557, and Meaningful Use Stage 3 represent policies that aimed to reduce barriers and expand access to gender-affirming care for covered entities (i.e., institutions that receive federal reimbursements and financial assistance for healthcare coverage) [45, 46]. Put another way, alongside the discrimination that transgender people experience in U.S. healthcare settings, the Affordable Care Act set forth federal and state policies to mitigate this discrimination and expand access to healthcare that is gender-affirming.

Although the ACA created new sources of healthcare for transgender people, funding for other basic resources have remained sparse. This gap includes housing, mental healthcare, food security, and, for medical institutions, includes funding to develop and maintain structural competency to address and eradicate embedded racism, sexism, ableism, and transphobia [47].

The third element of the outer context also shared by both cases is the COVID-19 pandemic. Although the impact is not entirely clear, the pandemic has likely limited transgender people’s access to healthcare afforded by the ACA. Similarly, it has likely encroached on access to other basic resources as well as funding for services related to those resources. TAP programming in Chicago as well as many services and medical education at UCLA moved to virtual environments in 2020–2021.

#### UCLA’s outer context

Initial funding for the Los Angeles Town Hall event was provided by the Tawani Foundation and the California HIV/AIDS Research Program through the UCLA Center for AIDS Research Health Disparities Core. Additional supplementary funding was provided by the UCLA AIDS Institute in order to implement the recommendations derived from data and analyses generated at the Los Angeles Transgender Town Hall. However, this funding was minimal and only sufficient to support very limited staff time as well as compensation for the participation of non-staff steering committee members. With the understanding that resources were limited, intervention options that were brainstormed for potential implementation were also limited.

#### TAP’s outer context

The TAP steering committee formed as a result of a grant award from the local department of public health and the Centers for Disease Control and Prevention. This grant called for community mobilizations to address structural racism in Chicago in order to expand access to HIV prevention and care for populations continuing to experience increasing incidence of HIV—specifically, Black and Latina transgender women, Black heterosexual cisgender women, and young Black and Latino men who have sex with men.

#### Inner Context: Organizational Characteristics, Culture, Leadership, & Fit

The inner settings of the two cases are difficult to characterize as the partner organizations represented by steering committee members in both cities varied widely in size, annual operating budgets, patient populations served, and scopes of services. In general, the institutional culture and leadership across the cases were supportive, and the intervention fit the values of the partner organizations well. However, the organizational and institutional characteristics supporting these efforts spanned a range of capacities, readiness for change, and receptive contexts to address transgender structural and individual vulnerability to poor health outcomes.

#### UCLA’s inner context

Since both the funding and the intervention was derived within UCLA and the David Geffen School of Medicine (DGSOM), UCLA is the focus of the inner context. The inner context consisted of a supportive leadership and culture at the David Geffen School of Medicine (DGSOM). The intervention was timely because when initial discussions of how to implement the recommendations occurred in 2017, UCLA DGSOM was in the early stages of a curriculum redesign that focused on topics related to health inequities and social justice. There was already early interest and momentum among several UCLA DGSOM faculty and leadership to address LGBTQ health and gender-affirming care. This synergistic fit between the values of DGSOM, its readiness for change, and the gender-affirming medical curriculum facilitated a receptive context for collaboration with the medical school and the rollout of the intervention.

#### TAP’s inner context

The inner context of TAP is constituted by and through the member organizations and their evolving relationships. Although the TAP steering committee’s core value of accountability seemed to align with each member organization’s mission and values, members of the steering committee did not always agree and requested several administrative discussions regarding transparency and equitable distribution of funding. These discussions, typically between leadership of member organizations, generated transparent financial shifts and also raised the issue of accountability for deliverables. While trust within the steering committee and across the partner organizations has grown, some member organizations have left the partnership for reasons ostensibly related to accountability and trust issues. At the same time, new partnerships with community-based organizations have formed, and new members have joined TAP’s steering committee. In addition, the small size and budgets of some partner organizations as well as the social marginalization many individuals on the steering committee experience daily have posed challenges to consistent and sustainable participation.

## Discussion

**Fig. 3:**
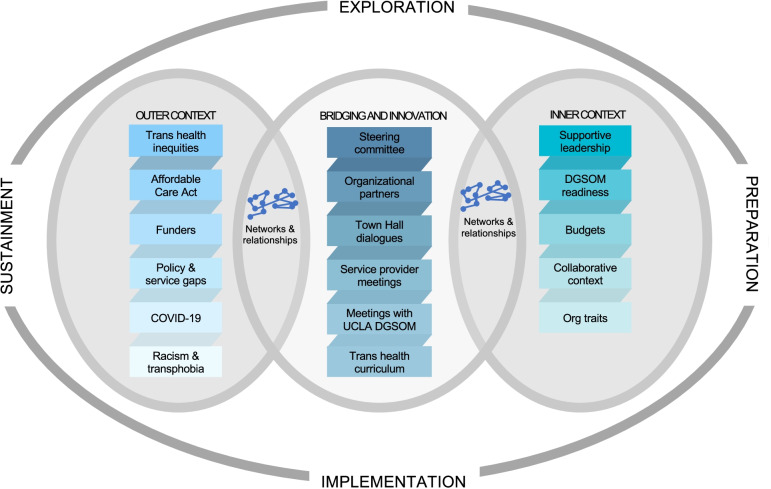
An adaptation of the EPIS framework for community-engagement and trans health equity; implementation of the UCLA CFAR Trans Health Project (2016–2021)

**Fig. 4:**
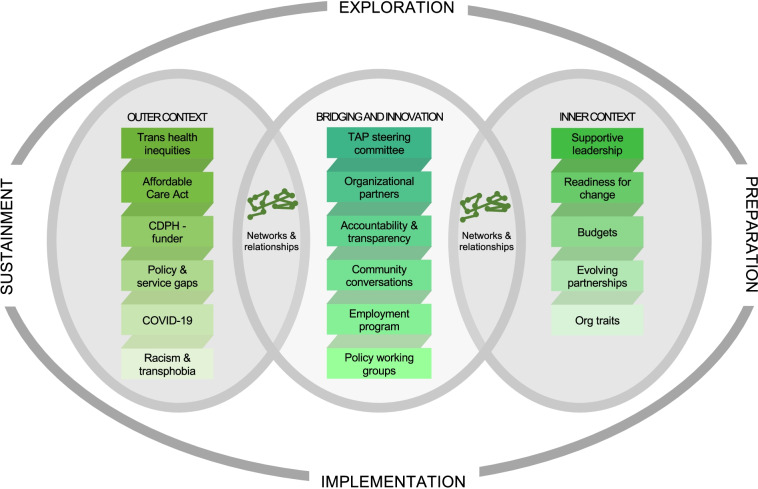
An adaptation of the EPIS framework for community-engagement and trans health equity; implementation of the Trans Accountability Project and the TAP Employment Pilot (2019–2021)

Adapting EPIS to describe and compare our exemplary cases [43] illuminated the ways in which differing levels of community engagement drove these multi-level interventions to address access to care and expand employment resources for transgender individuals and communities in Los Angeles and Chicago, respectively. The exploration, planning, design, and implementation of interventions to improve transgender health equity required input, guidance, and participation from trans communities. Considering the similar starting points and the divergent interventions that emerged from the work of the two steering committees, it is clear that there are numerous inflection points for community engagement but also for meaningful intervention. We propose an implementation framework for transgender health equity to guide future intervention development, implementation, dissemination, and adaptation (See Figs. 3 and 4**)**. Our exemplary cases of transgender community engagement as an implementation strategy bring into relief key insights that we transform into recommendations here.

### Bridging & Innovations: community engagement and steering committees

Although each case developed innovative interventions and bridges to extend organizational infrastructure and professional networks, the steering committee model and community engagement strategy lie at the root of bridging and innovation here. A steering committee of key stakeholders [48]—either entirely or in part from transgender communities as well as from community-based or stakeholder organizations—does not simply provide posthoc reflection or opportunities for prototyping with the priority population as a community advisory board might. Rather, a steering committee directs key aspects of intervention characteristics and scope. The professional relationships and community networks fostered by the steering committee between partner organizations and between trans and cisgender stakeholders also drive equity across inner and outer settings and help ensure trans health equity is centered and elevated as intervention development, innovation, and implementation unfold at different levels. Further, in Chicago, the core value of accountability and the distribution of funding across partner organizations scaffolded expanding professional networks with capacity building and infrastructure.

Implementation of interventions that expand access to care for marginalized populations often encounter barriers related to provider bias and discrimination, patient and community mistrust of medicine, and geographic inaccessibility [36, 49]. Both steering committees helped circumvent these potential barriers with the recognition of their respective intervention capacities but also by including a range of transgender voices to participate in assessment design, data collection and analysis, and the co-creation of subsequent intervention development and implementation. In the case of Los Angeles, the transgender panel and small group discussion components of the UCLA gender-affirming curriculum highlighted transgender perspectives and their unique experiences navigating healthcare. These narratives provided concrete examples of various ways that good intentions can be experienced as provider bias, gatekeeping, and discrimination; alternatively, they also illuminated how an intersectional lens can mitigate those biases and provide a basis of solidarity or alliance – between a cisgender provider and a transgender patient who are both immigrants, for example.

Aligned with recommendations of a more recent national study [12], the TAP steering committee determined that employment should be the focus of their intervention in order to impact upstream forces that limit Black and Latina transgender women’s access to healthcare. In the U.S., employment-based health insurance and stable incomes are critical for access to quality healthcare in addition to other key resources like housing. The innovation is that the steering committee and the employment intervention operate at multiple and intersectional levels: employment and healthcare policy, community networks and organizational infrastructure, and individual skills building via TAP Employment Program pilot. Specifically, the TAP steering committee’s intervention foci span structural, community, organizational, and individual levels: 1) state-, local-, and organizational-level employment policies, 2) network and community infrastructure-building, and 3) the employment skills and training of Black and Latina transgender women. This community-engaged, intersectional approach of the TAP steering committee is a novel way to improve transgender health equity.

### Inner and Outer Contexts: building organizational, community, and funder capacity

Our cases highlight why transgender community-engaged intervention development and implementation require substantial investments in time, organizational readiness, receptiveness, capacity building, and leadership development. In both cases, the outer context consists of sparse sources of funding and vast barriers to health equity including COVID-19. For the inner context of both cases, the partner organizations differed vastly in size, capacity, and annual operating budgets. Although the grants funding these two cases were both tied largely to HIV prevention dollars, the Chicago case had a far larger grant and longer grant duration compared to the Los Angeles case. However, when dispersed across four partner organizations in Chicago with numerous deliverables each year, the funding felt less impactful. Both COVID-19 and the trust and accountability issues may have impacted the low enrollment of the TAP pilot. Nonetheless, professional networks and capacities across organizations expanded and new collaborations emerged; for example, two TAP steering committee members collaborated with additional colleagues with clinical and administrative expertise to develop and pilot a group-level mental health intervention.

Currently emergent for both cases is sustainment, a phase not fully achieved nor analyzed in the results. The work of the UCLA project team and the trans health curriculum continue in part because the intervention aligned with goals and culture of UCLA DGSOM faculty and leadership. Although funding for the project through its original sources has ended, one of the co-authors continues to work with UCLA DGSOM to ensure that transgender health and gender-affirming care are fixtures of the curriculum. They are also working with UCLA DGSOM collaborators to share lessons through papers and conference workshops to help other universities and health systems develop similar models to improve training and education in those environments.

In Chicago, the TAP steering committee secured two additional years of funding to continue to build organizational, community, and individual capacity and to advocate for policy change. To extend the life of the steering committee further, TAP and the city of Chicago could adopt the model of the City and County of San Francisco whereby a transgender steering committee sits in and is funded by the department of public health. This restructuring might also mitigate some of the mistrust between partner organizations and the lead organization of the steering committee, enabling broader participation and coordination.

As others have documented, the current structure of universities, research, and funding for social services has severely constrained possibilities for accountable and impactful community engagement vis-à-vis implementation practice or research [50–53]. To promote trans health equity, traditional philanthropic and federal funding mechanisms may require restructuring to support community engagement during the pre-award and monitoring phases of a grant cycle and to facilitate more flexible and collaborative transgender community partnerships for grantees. As implementation scientists have noted in other domains [54, 55], grant requirements should be clearly aligned with the goals of trans health equity; new funding streams might operationalize trans inclusion, leadership development, capacity building at specified levels not only on the grantee teams but in funder study sections and program offices. Shared resources such as grant writing trainings, proposal templates, participatory budgeting, and submission infrastructure for award applications are needed so that transgender community partners can participate in the pre-award process more equitably [54, 55].

### Limitations

This study has several limitations. The two cases had very different funding parameters, budgets, and timelines for deliverables; consequently, the steering committee structures and organizational partnerships differed as did the interventions themselves. As a comparative case study, there were no quantifiable measures to benchmark or validate. Although these findings cannot be generalized, the themes resonate broadly across trans communities in the U.S. Transgender underemployment, in general, and underrepresentation in IS, caring professions, public health, and academia writ large, may be associated with the lack of evidence-based, trans-focused interventions and the lack of advancement in trans health equity via implementation practice and scientific research. We also cannot measure the downstream effects, for example, of the gender-affirming medical care curriculum that has grown over the last four years at UCLA.

## Conclusion

Our comparative case study demonstrates that an IS framework for trans health equity that employs a strategy of community engagement can provide guiding principles and, with additional resources and praxis, quantifiable benchmarks, and validated measures of trans health equity. Trans knowledge and experiences – from community members, scholars, advocates, funders, and activists emanating from the intersections of trans health inequities – are critical touchstones to achieve trans health equity.

## Data Availability

Data from the 2019 Trans Accountability Project community conversations/design workshops and stakeholder listening sessions are analyzed in Thompson HM, Hernandez E, Ortiz R, Ebosele I, Skora S, Beltran D, & Baker A. The Trans Accountability Project: Community Engagement to Address Structural Marginalization and Health Inequities. Prog Community Health Partnersh. Pre-print published online Feb 27, 2022; for more information about the employment intervention pilot data, contact Hale Thompson (hale_thompson@rush.edu). For more information concerning UCLA gender-affirming medical curriculum evaluation data or the recommendations of the steering committee, please contact Allison Clement (aclement@mednet.ucla.edu).

## Abbreviations

COVID-19: Coronavirus Disease 2019
EPIS: Exploration-planning-implementation-sustainment
HIV: Human immunodeficiency virus
IS: Implementation science
LGBTQ: Lesbian, gay, bisexual, transgender, and queer
TAP: Trans Accountability Project
UCLA: University of California-Los Angeles
CFAR: Center for AIDS Research
DGSOM: David Geffen School of Medicine
WCM: World Café model

## Footnotes

1 We use the term trans and transgender interchangeably. We also use the term Latina/x when referring to transfeminine people or research about Latin or Hispanic-descended transgender women that may include a spectrum of persons assigned male at birth who identify with a gender other than the one associated with male; in Chicago, some steering committee members who are transgender women and Latina, reject the term Latinx for themselves, but others do not. We also use the term Latinx when referring to trans people or research that includes both assigned-male and assigned-female at birth trans people of Latin or Hispanic-descent. We do not assume to know the identities of any of these individuals but are attempting to use the most inclusive language for social categories in research studies or in our own research described here.
